# Supplementary material for: High Arctic channel incision modulated by climate change and the emergence of polygonal ground
Source: Nat Commun. 2023 Sep 12;14:5297. doi: 10.1038/s41467-023-40795-9 (PMC10497538; doi:10.1038/s41467-023-40795-9)

Supplemental Information for:

"High Arctic channel incision modulated by climate change and the emergence of polygonal ground"

Date: July 28, 2023

Authors: Shawn M. Chartrand[1,2,*], A. Mark Jellinek[2], Antero Kukko[3], Anna Grau Galofre[4], Gordon R. Osinski[5] and Shannon Hibbard[5,6]

Affiliations:

1. School of Environmental Science, Simon Fraser University, Burnaby, BC, V5A 1S6, Canada
2. Department of Earth, Ocean and Atmospheric Sciences, University of British Columbia, Vancouver, BC, V6T 1Z4, Canada
3. Department of Remote Sensing and Photogrammetry, Finnish Geospatial Research Institute, National Land Survey of Finland, Espoo, 02150, Finland
4. Laboratoire de Planétologie et Géosciences, CNRS UMR 6112, Nantes Université, Le Mans Université et l'Université d'Angers, Nantes, 44322, France
5. Department of Earth Sciences, University of Western Ontario, London, ON, N6A 5B7, Canada
6. Jet Propulsion Laboratory, California Institute of Technology, Pasadena, CA, 91011, USA

Summary:

Here we refer to two movies, and provide additional results and figures from extended data analysis to support our discussion of polygon influence in shaping drainage network development and growth in Muskox Valley, Axel Heiberg Island. Extended data and results provided here consist of three tables and six figures. The tables document information related to the sediment mobility analysis completed using the traditional Shields method, coordinates for additional locations on Axel Heiberg Island which have similarities to the features discussed for Muskox Valley, and information related to estimates of peak flows calculated for proposed lake outburst floods. The figures document additional hydrodynamic modeling results, on the ground conditions related to a recent higher lake elevation, and active layer detachment slide and coarse sediment deposits along the main valley river channel, the full scanned 1959 aerial photograph and maps showing the locations of sites which show similarities to Muskox Valley. Cited references and figures are found in the main text.

Description of Supporting Movies

Active Layer Detachment Slide Seepage Flow Movie: See Supplementary Movie 1. Time-lapse movie captured over approximately 15 minutes at coordinates 79.372363° N and 87.808110° W. The movie shows the evolution of an active layer detachment slide face during a relatively large air temperature excursion in July 2019. On the day the movie was captured maximum air temperatures approached 20° C. The movie shows 1-10 centimeter-scale water rivulets draining from the thaw slump face in pulses corresponding to similarly small-scale mechanical failures and collapses of the soil profile. This released water carries sand, silt and clay to the slump base and drives rapid channel incision down stream The thaw slump face in the video measures approximately 4 m in height.

Polygon Trough Flow Movie: See Supplementary Movie 2. Walking movie captured on Devon Island at coordinates 75.489586° N and 89.887039° W. Movie shows water flow out of a glacial lake into a series of interconnected polygon troughs and flowing down gradient within the troughs. The spatial pattern of flow is non-uniform and decelerates and accelerates as flows move through the polygon troughs. Flows were not large enough in magnitude to transport bedload on the day of observation. However, the movie importantly illustrates a direct connection between the development of surface flows, and how it moves through the landscape within a periglacial and permafrost environment, supporting observations reported here.

Supplementary Table 1: **Riverbed particle mobility analysis within the lidar coverage area of muskox valley for plausible lake outburst peak flood conditions based on field observations**. Estimated mobility condition shown for the D_{50} and D_{90} particle sizes, with flows depths of 1 and 2 m, and flow top widths of 2 and 3 m. See Fig. 6 for estimated active layer thaw depths using the D_{90} particle size for scale.

TABLE ED1: ESTIMATED CRITICAL FLOW CONDITIONS FOR RIVERBED SURFACE PARTICLES

Depth *m*	Width *m*	D_{50} *m*	D_{90} *m*	$((\rho_s/\rho_w)-1)D_{50}$ *kg/m²*	$((\rho_s/\rho_w)-1)D_{90}$ *kg/m²*	t* D_{50}	t* D_{90}	t* critical *Lamb et al., 2009*	Velocity *m/s*	**Mobile?**	
1.0	2.0	0.020	0.096	0.03	0.15	0.23	0.05	0.062	1.2	**yes**	**no**
1.0	3.0	0.020	0.096	0.03	0.15	0.28	0.06	0.062	1.4	**yes**	**no**
2.0	2.0	0.020	0.096	0.03	0.15	0.31	0.07	0.062	1.5	**yes**	**yes**
2.0	3.0	0.020	0.096	0.03	0.15	0.40	0.08	0.045	1.8	**yes**	**yes**

Notes:

1. Section-averaged velocity estimate made using Manning Equation with a roughness of 0.055.
2. Local stream slope of 0.03 used for mobility calculation, and is based on LiDAR average slope of valley bottom.
3. Section shape factor of 0.5 used to modify flow area estimate for mobility calculation.
4. Particle density of 2600 kg/m3 and water density of 1000 kg/m3 assumed for mobility calculations.

Supplementary Table 2: **Coordinates of valleys and channels with similar characteristics to Muskox Valley**. Coordinates are UTM projection, Zone 16, and generally listed from north to south as shown in Supplementary Fig. 5.

UTM Zone 16

FID	Y_UTM	X_UTM
0	8867510	460908
1	8873870	458119
2	8873820	458544
3	8874220	458199
4	8875550	456527
5	8875330	457345
6	8877720	457010
7	8877490	457276
8	8877720	457461
9	8877500	456729
10	8878010	459746
11	8878760	459040
12	8878890	459345
13	8878780	459547
14	8880280	458849
15	8878660	461528
16	8879510	464081
17	8879280	464363
18	8881170	463206
19	8881690	465681
20	8881230	466911
21	8884870	467765
22	8894880	447162
23	8894770	446919
24	8894450	446497
25	8895130	445590
26	8895090	444386
27	8893360	442928
28	8891710	442694
29	8895150	438283
30	8895760	437970
31	8903820	443814
32	8903620	444144
33	8904730	444111
34	8906520	445420
35	8907080	446124
36	8912790	445730
37	8924540	446636
38	8927110	431791
39	8924710	428671
40	8894680	431759
41	8871340	466551

Supplementary Table 3: Estimated peak flow rates for the proposed outburst flood. Peak flow rates estimated using an empirical relationship for a dam breach[49]. See Methods in the main text for description of the empirical relationship. Values in the table are provided in imperial units for the calculation; the only parameter varied in the calculations was the breach hydraulic head, which is given in units of m at the left of the table.

TABLE ED3: ESTIMATED PEAK FLOW FOR LAKE OUTBURST FLOOD IN MUSKOX VALLEY

Breach Hydraulic Head	W	H	Tau	A	S.A	Peak Flow	
m	*f*	*f*	*hr*	*f*	*acres*	*cfs*	*cms*
0.5	9.8	1.6	0.167	45.2	19.0	63.21	**1.8**
1.0	9.8	3.3	0.167	45.2	19.0	177.75	**5.0**

Notes:

1. W: breach width; H: breach hydraulic head; Tau: breach failure time; A: relative breach length scale; S.A.: surface area of lake.

2. Estimated lake outburst flood peak flow rate from: Fread, D.L., 1981, Some Limitations of Dam-Breach Flood Routing Models, ASCE Fall Convention, St. Louis, MO, October 26-30, 1981.

Supplementary Fig. 1: Water runoff routing within Muskox Valley. Spatial distribution of locally-averaged downstream velocity for streamflows of 0.25 (top) and 5.0 (bottom) cms along the valley floor from station 900 to 1400m (Fig. 2a). Water runoff in the polygon region is spatially organized within interconnected polygon troughs whereas within the wetland region runoff spreads across the surface. See Methods of the main text for description of model setup. The simulations were conducted using BASEMENT-Basic Simulation Environment, ETH Zurich[52]. Satellite image sources: Esri | Maxar (DigitalGlobe) | Earthstar Geographics | GIS User Community, Imagery ID: 10df2279f9684e4a9f6a7f08febac2a9

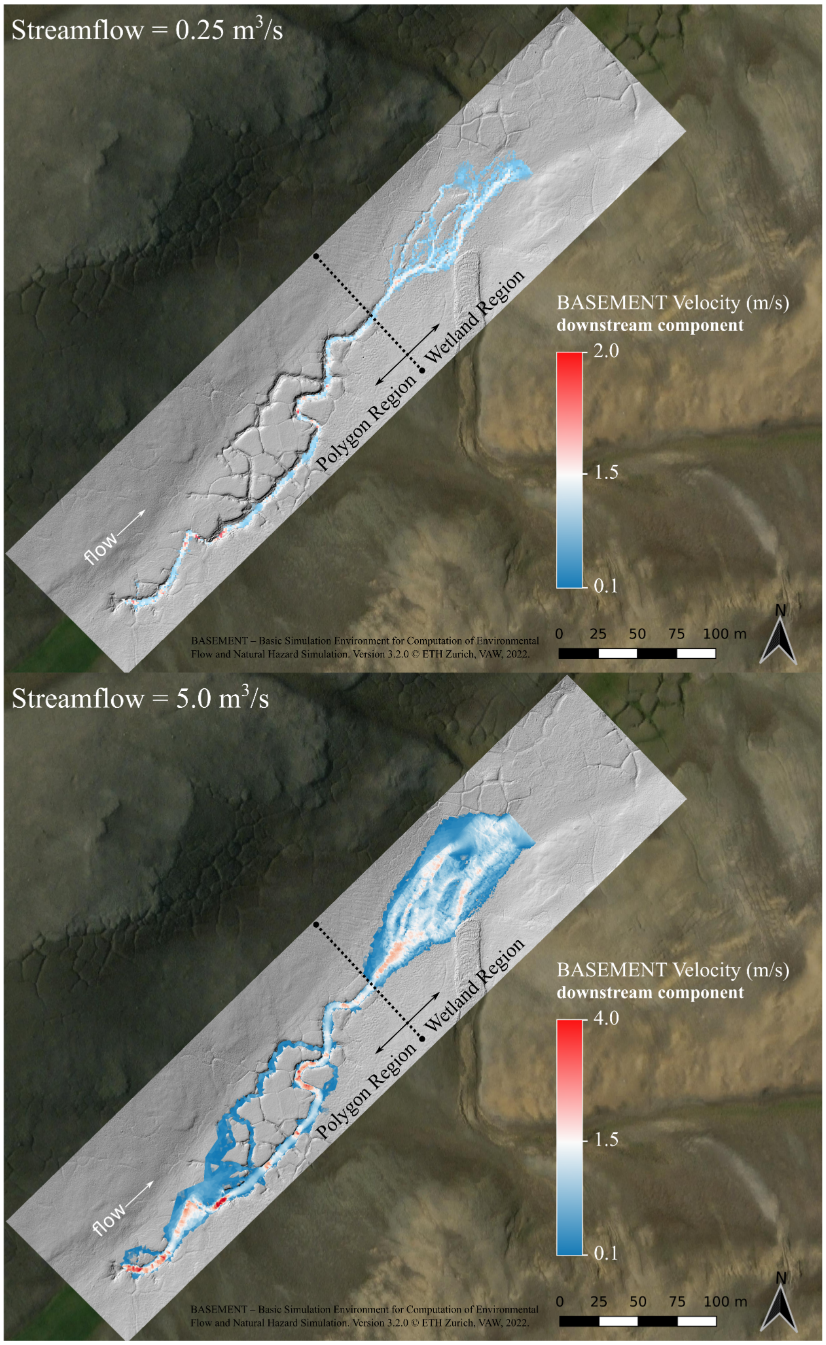

Supplementary Fig. 2: Supplemental Muskox Valley photographs. Hillshade is the same as shown in Fig. 2. The images on the right hand side were taken by authors and are color keyed with arrows pointing to the location and showing the general camera orientation of the photographs. The top image on the right is looking upstream at the lake. Arrows point to the abandoned shoreline hypothesized to mark the lake level prior to an outburst flood event which occurred sometime after 1959. Bottom left image looking to the south at the nose of an earthflow (see Fig. 2). Bottom right image looking at an instream gravel and cobble bar deposited by flood events capable of mobilizing bed surface sediments. Satellite image sources: Esri | Maxar (DigitalGlobe) | Earthstar Geographics | GIS User Community, Imagery ID: 10df2279f9684e4a9f6a7f08febac2a9.

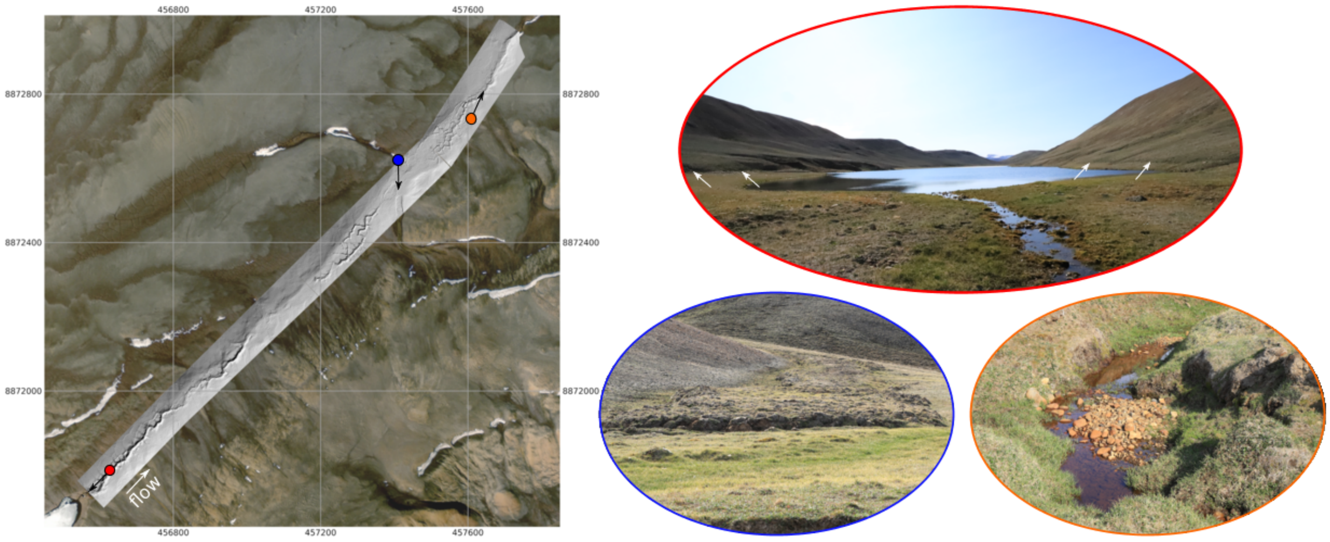

Supplementary Fig. 3: Supplemental Muskox Valley photographs. Hillshade is the same as shown in Fig. 2. The images on the right hand side were taken by authors and are color keyed with arrows pointing to the location and showing the general camera orientation of the photographs. The top image on the right is looking obliquely at the lake. Arrows point to the abandoned shoreline hypothesized to mark the lake level prior to an outburst flood event which occurred sometime after 1959. Bottom left image looking to the southwest along a channelized section with a fine gravel bar in the lower center. Bottom center right image looking to the southwest along a channelized section with a cobble and gravel bed. Bottom right image looking to the southwest along a channelized section also with a cobble and gravel bed. Grains in both the bottom left and right images show imbrication. Satellite image sources: Esri | Maxar (DigitalGlobe) | Earthstar Geographics | GIS User Community, Imagery ID: 10df2279f9684e4a9f6a7f08febac2a9.

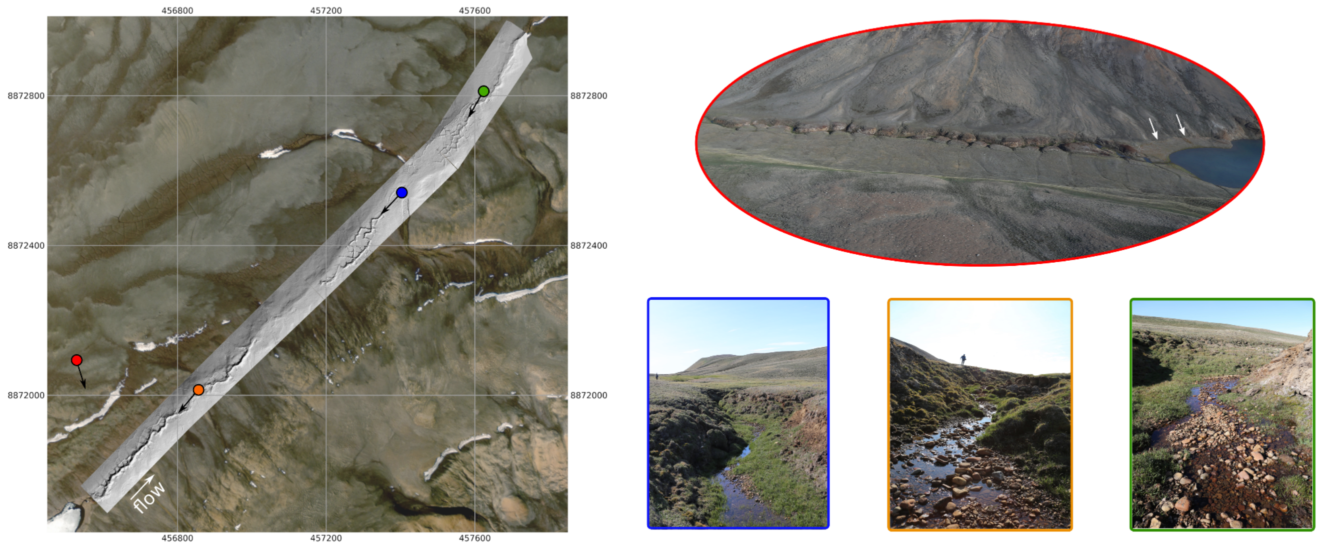

Supplementary Fig. 4: Muskox Valley in 1959. Limits of the inset image shown in Fig. 4 depicted by the dashed black outlined box. Muskox Valley proper follows the central, to lower-central part of the image. Image source from National Air Photo Library of Canada, roll and image #A16860_042; copyright holder: Natural Resources Canada.

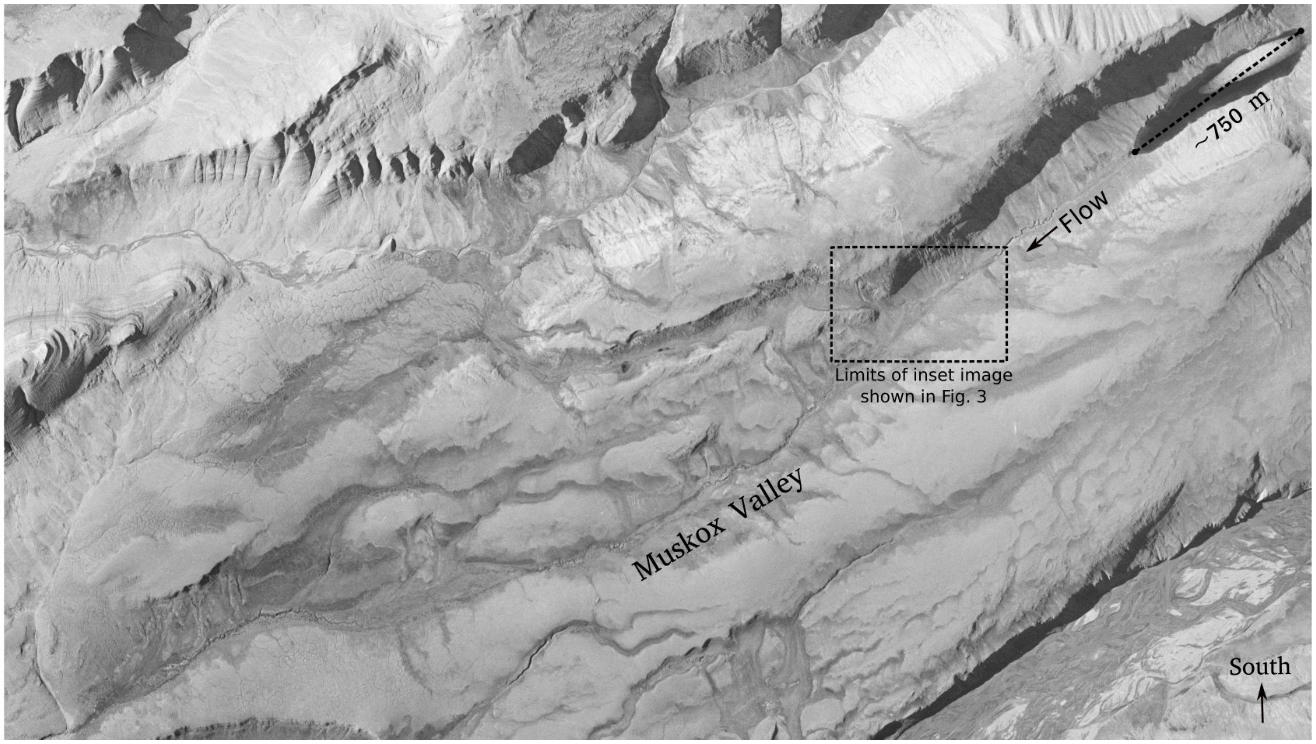

Supplementary Fig. 5: Valleys and channel systems similar to muskox valley. Map shows the location of valley and channel systems which share similar characteristics to those observed in Muskox Valley. Valleys marked by the green-colored dots are further detailed in panels a-d. See Supplementary Table 1 for coordinates of locations shown. **a**. The polygonal-influenced channel inherits topography from a lateral meltwater channel and receives water from supraglacial drainage. **b**. Channel that co-evolved with polygons and receives drainage from snowmelt, rainfall and seepage flow. **c**. The polygonal-influenced channel explores a subglacial channel (confirmed with a site visit), and receives water from snowmelt, rainfall and seepage flow. **d**. Polygons and channel system within a region where bedrock is expressed at the surface with steeply dipping strata which may impart control on post-glacial landform evolution. Water sources are snowmelt, rainfall and seepage flow. Satellite image sources for panels a-d: Esri | Maxar (DigitalGlobe) | GeoEye | Earthstar Geographics | CNES/Airbus DS | USDA | USGS | AeroGRID | IGN | GIS User Community; satellite image sources for the overview image same as panels a-d, with the addition of: AEX | Getmapping | IGP | swisstopo.

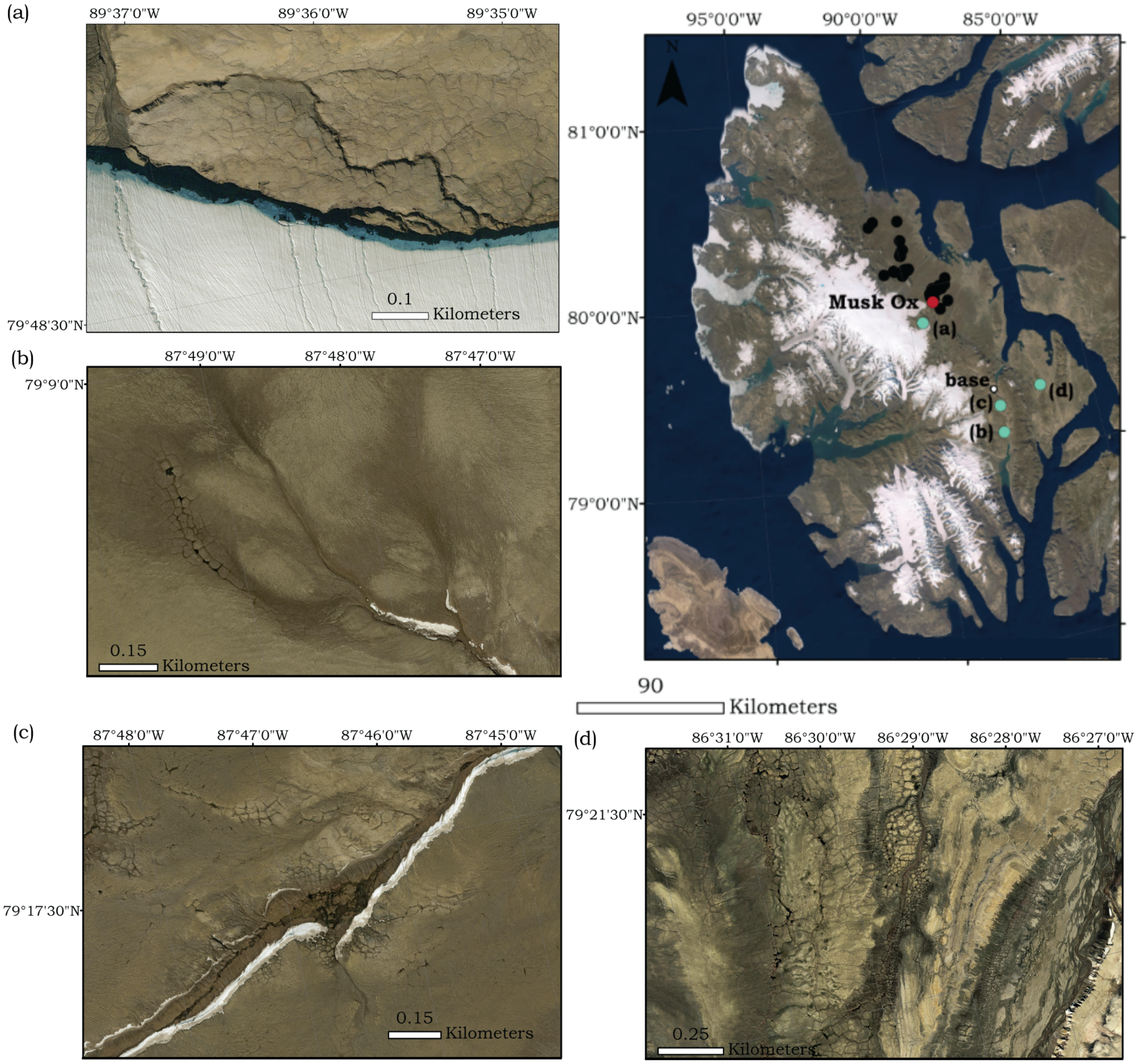

Supplementary Fig. 6: Illustration of width identification for width-depth ratio calculation. Example of cross-section extraction from rasterized LiDAR data, with illustrated profile location marked with a red arrow within the inset image of the bottom panel. The top panel shows elevation vs. distance. From this data, slope (middle panel) and curvature (bottom panel) were extracted. Curvature maximum and minimum values (bottom panel) mark the changes in slope and thus the boundaries of the channel, and thus reflect the top width, conditioned by negative and positive slope values respectively (see bottom and middle channels). Bottom width, measured using the same technique and shown in the figure but not reported, is used as means of an internal consistency check (top width> bottom width). Method used previously for similar calculations focused on subglacial channels[48]. Satellite image sources for lower inset: Esri | Maxar (DigitalGlobe) | Earthstar Geographics | GIS User Community, Imagery ID: 10df2279f9684e4a9f6a7f08febac2a9; Colorized hillshade corresponds to our LiDAR raster.

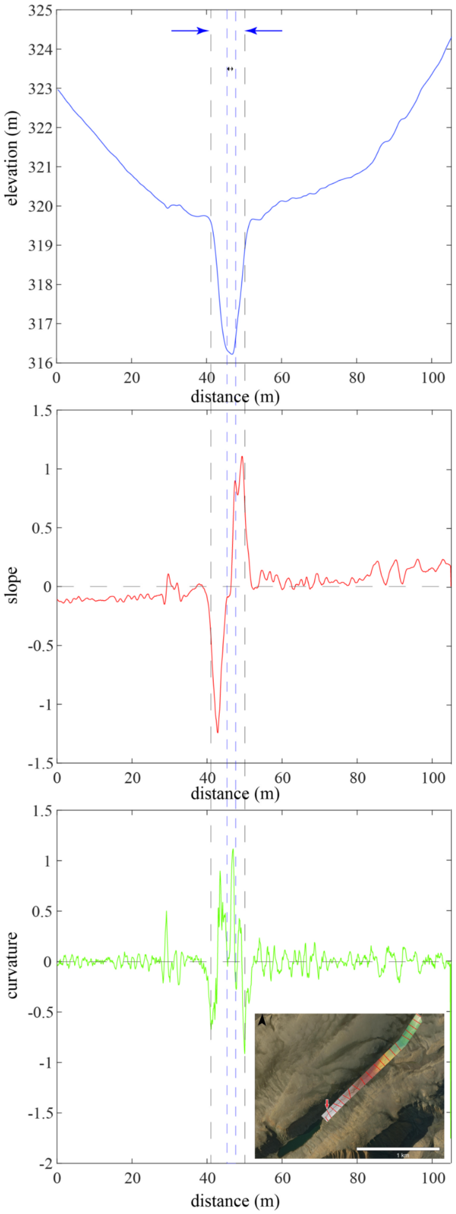

Supplement: Supplementary file 1 — Supplementary Information [file 41467_2023_40795_MOESM1_ESM.pdf]
